# Direct and indirect assessment of functional abilities in patients with Parkinson’s disease transitioning to dementia

**DOI:** 10.1590/1980-57642020dn14-020011

**Published:** 2020

**Authors:** Gisele Saraiva Reis de Oliveira, Lúcia Bressan, Fernanda Balarini, Raquel Silveira Jesuino e Silva, Manuelina Mariana Capellari Macruz Brito, Maria Paula Foss, Bruno Lopes Santos-Lobato, Vitor Tumas

**Affiliations:** 1 Department of Neuroscience and Behavior, Ribeirão Preto Medical School, University of São Paulo, Ribeirão Preto, SP, Brazil.

**Keywords:** Parkinson’s disease, dementia, mild cognitive Impairment, functional assessment, doença de Parkinson, demência, avaliação funcional, avaliação direta da capacidade funcional, questionário de pílulas

## Abstract

Cognitive impairment is common in patients with Parkinson’s disease (PD), and evaluation of functional abilities is crucial for diagnosis of dementia.

**Objective:**

We evaluated differences between direct and indirect functional assessment methods to evaluate functional abilities in PD patients.

**Methods:**

We evaluated 32 patients with PD and suspected mild dementia using direct and indirect assessment methods.

**Results:**

There was a significant difference between the scores of direct and indirect methods of assessment. Patients and close informants usually overestimated their abilities in many ADL. However, all functional assessment tools used in this study had a relatively good accuracy to predict abnormal performance in a global cognitive scale. Patients with normal cognition according to scores in a global cognitive scale may have some functional impairment in ADL. Direct Assessment of Functional Ability (DAFA) scores correlated linearly with scores in global cognitive scales, and especially with scores in the domains of memory and concentration.

**Conclusion:**

Patients and close informants usually overestimate their instrumental abilities in ADL. The direct assessment of daily functioning was more reliable than indirect tools to assess functional losses in patients with PD. Finally, some patients with PD but no dementia may present functional losses in ADL.

## RESUMO.

O comprometimento cognitivo é comum em pacientes com doença de Parkinson (DP), e a avaliação das habilidades funcionais é crucial para o diagnóstico de demência.

**Objetivo:**

Avaliamos diferenças entre os métodos de avaliação funcional direta e indireta para avaliar habilidades funcionais em pacientes com DP.

**Métodos:**

Foram avaliados 32 pacientes com DP e suspeita de demência inicial usando métodos de avaliação direta e indireta.

**Resultados:**

Houve uma diferença significativa entre os escores dos métodos diretos e indiretos de avaliação. Pacientes e informantes geralmente superestimaram suas habilidades em muitas atividades da vida diária (AVD). No entanto, todas as ferramentas de avaliação funcional utilizadas neste estudo tiveram uma precisão relativamente boa para prever desempenho anormal em uma escala cognitiva global. Pacientes com cognição normal, de acordo com os escores em uma escala cognitiva global, podem apresentar algum comprometimento funcional nas AVD. As pontuações do *Direct Assessment of Functional Ability* (DAFA) correlacionaram-se linearmente com as pontuações nas escalas cognitivas globais e, especialmente, com as pontuações nos domínios da memória e concentração.

**Conclusão:**

Pacientes e informantes próximos geralmente superestimam suas habilidades instrumentais nas AVD. A avaliação direta do funcionamento diário foi mais confiável do que ferramentas indiretas para avaliar perdas funcionais em pacientes com DP. Finalmente, alguns pacientes com DP, mas sem demência, podem apresentar perdas funcionais nas AVD

Cognitive impairment is a common feature of Parkinson’s disease (PD). Evidence shows that about 25% of PD patients present mild cognitive impairment (MCI) and 30% present dementia (PDD), while 80% of patients are expected to develop dementia after 20 years of disease duration.^1^^-^ 3 The diagnosis of PDD is associated with impaired quality of life of patients and caregivers, increased risk of institutionalization, and lower life expectancy.^4^^-^ 6 Recently, a task force of the International Parkinson and Movement Disorders Society (IP-MDS) operationalized the diagnostic criteria for PDD and MCI in PD.^7^^-^ 9 However, uncertainties remain, such as the best evaluation tool for diagnosing functional decline resulting from cognitive impairment in PD.^9^

The PDD diagnostic criteria require that cognitive dysfunction be severe enough to impair daily life, independently of impairments related to motor or autonomic symptoms.^7^ Defining the impact of cognitive loss on activities of daily living (ADL) therefore represents an important step in the diagnosis of cognitive dysfunction in PD, as in other dementias.

Self-reported and informant-based methods of functional assessment are the main resources used to define functional impairment associated with cognitive loss. However, ability estimates by patients and close informants can be imprecise, and it may be difficult to discriminate if the source of impairment is related to cognitive or motor symptoms.^10^ These issues are critical when evaluating patients transitioning to or with mild dementia. For patients with PD and cognitive loss, the application of performance-based assessments of ADL is likely to be more precise than information-based assessments to define functional abilities.^11^ Objective methods allow for the direct observation of the patient’s abilities to perform basic or instrumental ADL and of the interference of motor symptoms in the execution of these tasks. To date, however, few studies have evaluated performance-based assessments of ADL in patients with PD.^9^

In this study, we evaluated methods of direct and indirect assessment of instrumental ADL in patients with PD and suspected dementia. Our aim was to evaluate both methods, and compare their validity for the diagnosis of functional loss due to cognitive impairment.

## METHODS

For this cross-sectional study, we invited patients with PD diagnosed according to specific criteria,^12^ consecutively attending the movement disorders outpatient clinic of the Ribeirão Preto Medical School Hospital between March 2013 and April 2014.

The inclusion criteria were: (1) age above 50 years; (2) stable therapeutic regimen; and (3) presence of cognitive complaints and abnormalities in the clinical evaluation suggesting the diagnosis of mild dementia. We based the suspicion of mild dementia on a clinical interview and through application of brief clinical tools. We assessed symptoms and signs of PD using the Movement Disorders Society - Unified PD Rating Scale (MDS-UPDRS),^13^ and performed a brief neuropsychological evaluation using the mini-mental state examination (MMSE) and the semantic verbal fluency test (VFT).^14^^,^ 15 The patient health questionnaire was used to screen for depression,^16^ while the instrumental abilities of patients were initially assessed using the “pill questionnaire”.^17^ Based on this examination performed by a clinical neurologist, if the patient scored 1 or 2 on item 1.1 of the MDS-UPDRS, but the clinician still had suspicions about the patient’s current cognitive state, they were invited to participate in the study.

The exclusion criteria were: (1) inability to remain in the “on state” to perform all clinical evaluations; (2) presence of severe systemic diseases or any other serious or uncontrolled neurological or psychiatric symptoms, including major depression; and (3) use of high doses of antidepressants, anxiolytics or anticonvulsants.

The Ethics Committee of the Ribeirão Preto Medical School Hospital, which observes the recommendations of the Declaration of Helsinki, approved the research project and all volunteers agreed to participate by signing an informed consent form (number of registration 9106/2008).

After inclusion in the study, a neuropsychologist interviewed patients and close informants, when available. A close informant was considered to be any companion whom the examiner considered able to reliably report on the patient’s functional abilities.

Abilities in ADL were assessed using Pfeffer’s functional activities questionnaire (PFAQ).^18^ When patients were assessed in the presence of an informant, the examiner defined the scores of PFAQ items by considering the worst score indicated, regardless of who defined this score (patient or informant). The PFAQ is an informant-based instrument comprising 10 items for assessing a variety of instrumental ADL grouped into seven functional domains (money management, shopping, hobbies, meal preparation, awareness, reading, and transportation). For each item, the examiner assigned scores between 0 (if the patient was able to perform the task alone without difficulties) and 3 (if the patient was not able to perform the task even with some assistance). The total score ranges from 0 to 30 and higher scores indicate poorer performance in ADL. In addition to the PFAQ, the neuropsychologist also performed a cognitive evaluation using the Mattis dementia rating scale (MDRS).^19^^,^ 20

An occupational therapist then performed an objective assessment of abilities in ADL using the Direct Assessment of Functional Ability (DAFA), which measures the same abilities assessed by the PFAQ. The DAFA is indicated for use in clinical settings to evaluate patients with mild to moderate dementia.^11^ In this assessment, the examiner directly observed the patient while they performed 10 tasks that reproduce the 10 items evaluated in the PFAQ. The scoring system of the DAFA is equivalent to that used in the PFAQ, and the total score ranges from 0 to 30. The occupational therapist also made an objective assessment of the patients’ functional abilities on the “telephone test” and the “pill test.”

On the telephone test, patients received a phone call with a message and then made a call to send the message forward. Performance on this task was assessed based on six different aspects: (1) answering the phone (picking up the phone and starting a conversation); (2) identifying the call (saying who made the call or giving references about it); (3) identifying the message; (4) making a phone call (picking up the phone); (5) making the call by dialing the number provided (verbally or in writing); and (6) passing the message on. Each item received a score of 0 (if the patient was able to perform the task alone), 1 (if the patient was able to perform the task, but only with some assistance) or 2 (if the patient was unable to perform the task even with assistance). The total score in the telephone test ranges from 0 to 12.

In the pill test, the examiner placed several antiparkinsonian drugs (pills and containers) on a table in front of the patient and asked them to identify the medications they used and to describe their treatment regimen. The investigator scored the patients’ performance as 0 (patient was able to describe drugs, doses and schedules spontaneously and clearly), 1 (patient required assistance from the examiner, but was able to describe the therapeutic scheme clearly and without errors) or 2 (patient was unable to describe the drugs or therapeutic scheme, even with help).

The clinical and demographic data of the sample were analyzed with descriptive statistics. Patients were classified as having normal or abnormal cognition according to MDRS and/or MMSE scores and normative data for the Brazilian population, adjusted for level of education.^14^^,^ 19 ^,^ 21 Spearman’s correlation test was used to investigate correlations between variables, and the Wilcoxon test for paired samples was employed to test for differences in scores. The rate of concordance of the scores on the two scales were also calculated (number of equal scores/total number of scores).

Scatter plots were produced to show the relationship between raw scores and ROC curve analysis was carried out to investigate the discriminant validity of the functional scales to predict abnormal scores on a global cognitive scale. The association between cognitive performance and functional abilities was analyzed using univariate analysis with adjustment for covariates.

All analyses were done using the Statistical Package for the Social Sciences (SPSS), version 17.0 (SPSS Inc., Chicago, USA) and the level of statistical significance used was p<0.05.

## RESULTS

A total of 40 consecutive patients were invited to participate, of which 8 were excluded because they did not complete all evaluations. Table 1 presents the clinical and demographic data of the final 32 patients with PD included in this study. Participants were predominantly male and had low education (mean of four years of education). Most were in stage II and none were in stages IV or V of the Hoehn and Yahr scale. The mean age was 69 years, all patients used antiparkinsonian drugs and almost all used levodopa. Most of the patients (62.5%) were evaluated in the presence of a close informant.

**Table 1:** Clinical characteristics of PD patients with suspected mild dementia.

Clinical characteristics		n=32
Age at assessment (years)^a^		69 (8.1)
Education (years) ^a^		4.5 (2.9)
Disease duration (years) ^a^		8.4 (6.6)
MDS-UPDRS Part 3 ^a^		14.12 (5.9)
MMSE score ^a^		23 (4.2)
MDRS score ^a^		122 (13.4)
DAFA total score ^a^		8 (3.3)
PFAQ total score ^a^		1.9 (3.3)
Pill test ^a^		45.7 (14.2)
Telephone test ^a^		3.3 (2.7)
Male sex, n (%)		27 (84.3)
Abnormal MMSE score, n (%)		3 (10)
Abnormal MDRS score, n (%)		4 (12.5)
Abnormal MMSE and MDRS scores, n (%)		6 (18.7)
Assessed with a close informant, n (%)		20 (62.5)
Use of levodopa, n (%)		27 (84.4)
Presenting wearing-off, n (%)		13 (40)
Presenting dyskinesia, n (%)		6 (19)
Hoehn and Yahr stage, n (%)	I	1
	II	23
	III	8
	IV and V	0

a Values expressed as mean (SD); DAFA: Direct Assessment of Functional Abilities; MMSE: Mini-Mental State Examination; MDRS: Mattis Dementia Rating Scale; PFAQ: Pfeffer's Functional Activities Questionnaire; MDS-UPDRS: Movement Disorders Society - Unified Parkinson's Disease Rating Scale.

The mean performance of patients on the two scales of global cognitive assessment was very close to the general cut-off scores used for the diagnosis of global cognitive deficit, namely, scores <123 on the MDRS and <26 on the MMSE.^22^ Thirteen of the 32 patients were classified as having abnormal cognition, because they had abnormal scores on at least one of the global cognitive scales (three patients had abnormal MMSE scores, four patients had abnormal MDRS scores, while six patients had abnormal scores on both scales).^14^^,^ 19

A significant difference was found between scores on the DAFA and the PFAQ (Table 2). Total scores on the DAFA were significantly higher than total scores on the PFAQ (p=0.0001). The scores for most items of the DAFA were significantly higher than the respective items of the PFAQ, except for items: 5 (preparing coffee), 6 (preparing a sandwich), and 9 (remembering appointments). The rate of concordance between scores on the DAFA and the PFAQ ranged from 0.18 to 0.83. The highest rates were observed for items: 4 (hobbies, 0.71), 5 (preparing coffee, 0.83), and 6 (preparing a sandwich, 0.65). This difference was significant even when analyzing the data separately, that is, considering the scores obtained only with the patient information, or information obtained with the help of a close informant.

**Table 2:** Comparison between DAFA and PFAQ scores in PD patients with suspected mild dementia.

Item of the scale	PFAQ	DAFA	p-value	Rate of concordance
1. Money management^#^	0.16 (0.45)	0.87 (0.97)	0.001*	0.46
2. Money management^#^	0.09 (0.39)	0.47 (0.62)	0.003*	0.56
3. Shopping	0.13 (0.42)	0.84 (0.92)	0.0001*	0.50
4. Hobbies	0.23 (0.43)	0.58 (0.89)	0.031	0.71
5. Meal preparation (coffee)	0.03 (0.18)	0.25 (0.57)	0.063	0.83
6. Meal preparation (sandwich)	0.16 (0.63)	0.34 (0.48)	0.183	0.65
7. Awareness	0.13 (0.49)	0.84 (1.17)	0.001*	0.59
8. Reading	0.22 (0.49)	1.91 (1.03)	0.001*	0.18
9. Awareness (appointments)	0.69 (0.86)	0.81 (0.78)	0.543	0.34
10. Transportation	0.06 (0.25)	1.16 (0.95)	0.001*	0.25
Total score	1.88 (3.13)	8.00 (5.44)	0.0001*	0.50

Values expressed as mean (SD);

* p <0.05, Wilcoxon test for paired samples; DAFA: Direct Assessment of Functional Abilities; PFAQ: Pfeffer's Functional Activities Questionnaire;

# Money management 1: assesses the patient's ability to write a check, Money Management 2: evaluates a patient's ability to fill out a form with personal information.

Scores were higher on the pill test than on the “pill questionnaire” (p=0.03). The rate of concordance between these scores was 0.53.

There was a significant, but low-to-moderate correlation (p=0.04, r=0.49) of total scores on the DAFA and PFAQ with scores on the pill questionnaire and pill test (p=0.007, r=0.47).

Total scores on the DAFA correlated significantly with MMSE scores (p=0.0001, r= -0.72), MDRS scores (p=0.0001, r= -0.73) and age (p=0.011, r= -0.44), but not with education (p=0.17). DAFA scores did not correlate with the MDS-UPDRS motor score (p=0.16) or with the Hoehn and Yahr scale (p=0.64).

Total score on the PFAQ correlated significantly with both MMSE scores (p=0.005, r= -0.48) and MDRS scores (p=0.048, r= -0.35), but not with age (p=0.88) or education (p=0.64).

**Figure 1:**
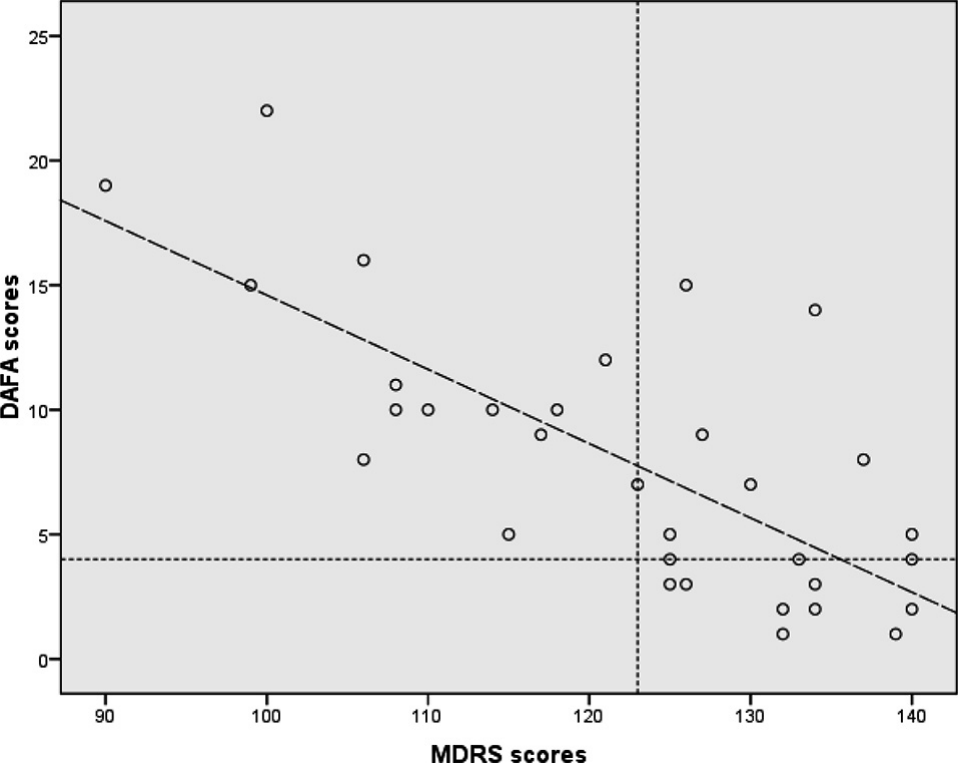
Scatter plot of patient scores on the Direct Assessment of Functional Ability (DAFA) and on the Mattis Dementia Rating Scale (MDRS). The dashed horizontal line represents the best DAFA cut-off score to predict an abnormal score on the MDRS. The dashed vertical line represents the best general MDRS cut-off score to predict dementia. The dashed sloped line represents the estimated regression line (linear regression equation - y = 44.4 - 0.298x).

Scatter plots showed a linear correlation between DAFA and MDRS scores (Figure 1). The regression coefficient for the effect of the MDRS on DAFA scores was 0.73 (p<0.001); when age was included in the model, the coefficient was 0.77 (p<0.001). When including age, education, and motor scores on the MDS-UPDRS in the linear regression model, the resulting coefficient was 0.77 (p<0.001). There was a linear correlation between MMSE and DAFA scores (coefficient=0.72; p=0.001).

DAFA scores correlated significantly with all subscores of the MDRS: attention (p=0.02, r= -0.40), initiative (p=0.001, r= -0.50), construction (p=0.02, r= -0.41), conceptualization (p=0.001, r= -0.72) and memory (p=0.001, r= -0.66). Scatter plots showed linear correlations between DAFA scores and all subscores of the MDRS. The regression coefficient for the effect of the memory subscore on DAFA scores was 0.68 (p=0.0001), while for the conceptualization subscore the coefficient was 0.51 (p=0.0001). Including memory and conceptualization subscores in the linear regression model resulted in a coefficient of 0.79 (p=0.0001). The inclusion of all five subscores of the MDRS in the model resulted in a coefficient of 0.82 (p=0.0001)

The ROC curve analysis showed that all functional assessment tools used in this study had relatively good accuracy for predicting abnormal performance on a global cognitive scale (Table 3). The two best tests were the “telephone test” and the DAFA, which had the best accuracy for characterizing abnormal performance on a global cognitive scale (areas under the curve of 0.83 and 0.76, respectively). In general, the best cut-off scores to identify abnormal cognitive performance were very low for all the instruments evaluated. The best discriminant cut-off score was higher for the DAFA than for the PFAQ.

**Table 3:** Results of ROC curve analysis considering ability of functional tests to predict abnormal performance on a global cognitive test (MMSE and/or MDRS).

Functional test	AUC	Predicts at least one abnormal cognitive score if score is greater than	Sensitivity	Specificity	PPV	NPV
Pill test	0.668	0	0.80	0.58	0.55	0.84
Pill questionnaire	0.637	0	0.46	0.63	0.56	0.85
Telephone test	0.837	2	0.82	0.63	0.56	0.85
DAFA	0.761	4	0.90	0.53	0.57	0.90
PFAQ	0.661	2	0.23	0.748	0.42	0.60

AUC: Area Under the Curve, DAFA: Direct Assessment of Functional Abilities, MDRS: Mattis Dementia Rating Scale, MMSE: Mini-Mental State Examination, PFAQ: Pfeffer's Functional Activities Questionnaire, PPN: Positive predictive value, NPV: Negative predictive value

## DISCUSSION

Our study assessed a small but representative sample of PD patients presenting with cognitive performance bordering on a diagnosis of dementia. Considering the hypothesis that patients with PD evolve in a gradual process of cognitive loss from the stage of MCI to dementia, our patients were close to a transition point between these two cognitive states. From a clinical perspective, these patients pose a challenge for cognitive diagnosis. Exploring aspects regarding the assessment of functional abilities, some interesting issues were noted.

Patients with PD and their informants tend to overestimate patients’ abilities in ADL. This was clear for most instrumental activities assessed, including money management, shopping, hobbies, reading, transportation, and medication management. However, for some tasks such as meal preparation and awareness, the overestimation of abilities was not so significant. The discordance between subjective self-reports and objective performance assessments of ADL has been previously described in PD patients and in other patients with dementia.^10^^,^ 11 ^,^ 23 ^-^ 25 There are probably many reasons for these discrepancies.^10^ Patients unawareness of their deficits translates to poor recognition of changes in ADL abilities, as described earlier for patients with Alzheimer’s disease.^26^ However, this hypothesis does not explain the poor estimates of functionality made by close informants. Close informants of Alzheimer’s patients tended to underestimate the patient’s functional ability, while in our study, close informants of PD patients tended to overestimate the patient’s functional abilities.^24^^,^ 25 The presence of motor dysfunction could be a factor affecting patient and informant insights regarding performance on a host of activities. However, our findings showed no relevant correlation between motor symptoms and performance or reports on ADL abilities. It is possible that aspects related to the validity of the instruments used to evaluate functionality are more relevant to this discussion. The precision of direct assessment methods can be affected by the fact that patients may perform differently at home than in other settings. Therefore, all methods used for functional evaluation should ideally estimate or measure abilities in tasks usually performed by the patient, where this may vary for each subject. As we observed, patients and informants provide more accurate reports on abilities to perform some tasks than others, and the reason for this was not clear in our study.

Despite the discrepancies between direct and indirect assessment methods, and the uncertainties about the validity of the scales, our study showed that the instruments used to evaluate abilities in ADL had good accuracy to predict abnormal performance on a global cognitive scale. The establishment of accurate and reliable methods to measure the functional abilities of patients with PD and cognitive losses is essential for an accurate diagnosis of dementia. Currently, there is no consensus about the best instrument for assessing functional decline due to cognitive impairment in PD. The recommendation of the MDS task force is the use of an unstructured interview on daily functioning or the “pill questionnaire”.^17^ Few studies have evaluated the diagnostic validity of the “pill questionnaire” and, although the findings available are controversial, some authors have considered the tool less accurate than expected when performing relevant assessments.^22^^,^ 26 Recently, two brief PD-specific functional questionnaires were designed to measure the impact of cognitive loss on ADL.^27^^,^ 28 However, knowledge about the validity and reliability of these scales remains incomplete. All the previously mentioned instruments are indirect assessments of ADL abilities. Some investigators hold that a direct assessment of functional abilities would be helpful to avoid bias in data obtained from patients and informants.^9^ The validity of a performance-based assessment of cognitive functional ability was only recently evaluated in PD.^29^

Our observations showed that, for the diagnosis of significant cognitive decline, both objective and subjective scales are useful, especially when their cut-off scores were well defined and validated. However, in our study, direct methods performed better than indirect methods of assessment. Furthermore, we found that simple performance-based tests, such as the telephone test and the pill test, had similar performance to extensive assessments such as the DAFA. We therefore concluded from our findings that the application of a simple, direct assessment of functional abilities could prove sufficient for use in clinical settings.

We found that scores on the DAFA correlated linearly with MDRS scores, and that there was a gradual decline in functional abilities with a decrease in cognitive score measured by the MDRS. Some patients with normal scores on a global cognitive scale had deficits detected by the DAFA. This suggests that patients with PD and without dementia may also present functional deficits, as described in previous reports.^22^^,^ 26 Patients with PD and MCI may have some loss in instrumental activities, and the definition of substantial functional deficit may, therefore, be somewhat arbitrary. Probably, the delimitation of a threshold for diagnosis of significant loss of functional abilities may be variable according to each patient, and the cognitive evaluation will be the key for diagnosis.

DAFA scores correlated with all MDRS subscores, but especially with the domains of conceptualization and memory. Most studies show a strong correlation between executive function and functional abilities, but our findings suggest that memory is also a relevant cognitive domain for the maintenance of functionality in patients with PD.

Our findings showed that instruments like the DAFA can serve as an outcome measure in follow-up of functional abilities. Most therapeutic trials use cognitive scales as their main outcome measures, but improvements in their scores may not reflect the recovery of functional abilities. Objective instruments to assess abilities in ADL, such as the DAFA, could be useful in the assessment of functional improvement resulting from therapeutic interventions.

In conclusion, patients and close informants generally overestimate their instrumental abilities in ADL. The direct assessment of daily functioning is more reliable than indirect methods for determining functional losses in patients with PD, and some PD patients without dementia may present some functional loss in ADL abilities.
